# Formulation Design, Statistical Optimization, and In Vitro Evaluation of a Naringenin Nanoemulsion to Enhance Apoptotic Activity in A549 Lung Cancer Cells

**DOI:** 10.3390/ph13070152

**Published:** 2020-07-15

**Authors:** Shadab Md, Nabil A. Alhakamy, Hibah M. Aldawsari, Mohammad Husain, Sabna Kotta, Samaa T. Abdullah, Usama A. Fahmy, Mohamed A. Alfaleh, Hani Z. Asfour

**Affiliations:** 1 Department of Pharmaceutics, Faculty of Pharmacy, King Abdulaziz University, Jeddah 21589, Saudi Arabia; nalhakamy@kau.edu.sa (N.A.A.); haldosari@kau.edu.sa (H.M.A.); skotta@kau.edu.sa (S.K.); uahmedkauedu.sa@kau.edu.sa (U.F.); 2 Advanced Drug Delivery Research Group, Faculty of Pharmacy, King Abdulaziz University, Jeddah 21589, Saudi Arabia; maalfaleh@kau.edu.sa; 3 Center of Excellence for Drug Research & Pharmaceutical Industries, King Abdulaziz University, Jeddah 21589, Saudi Arabia; 4 Department of Biotechnology, Jamia Millia Islamia (Central University), New Delhi 110025, India; mhusain2@jmi.ac.in; 5 Department of Biological Sciences-Cancer Immunology, Faculty of sciences, King Abdulaziz University, Jeddah 21589, Saudi Arabia; stheebabdallah@stu.kau.edu.sa; 6 Department of Natural products and alternative medicine, Faculty of Pharmacy, King Abdulaziz University, Jeddah 21589, Saudi Arabia; 7 Department of Medical Microbiology and Parasitology, Faculty of Medicine, King Abdulaziz University, Jeddah 21589, Saudi Arabia; hasfour@kau.edu.sa

**Keywords:** apoptosis, Box–Behnken design, naringenin, natural product, lung cancer

## Abstract

Naringenin (NAR), a flavonoid mainly found in citrus and grapefruits, has proven anti-cancer activities. However, the poor water solubility and low bioavailability of NAR limits its use as a therapeutic agent. The aim of this study was to develop and optimize stable naringenin nanoemulsions (NAR-NE) using a Box–Behnken experimental design to obtain a formulation with a higher efficiency. Anticancer activity of optimized NAR-NE was evaluated in A549 lung cancer cells using cell viability, flow-cytometric assays, and enzyme-linked immunosorbent assay. The stabilized nanoemulsion, which showed a spherical surface morphology, had a globule size of 85.6 ± 2.1 nm, a polydispersity index of 0.263 ± 0.02, a zeta potential of −9.6 ± 1.2 mV, and a drug content of 97.34 ± 1.3%. The NAR release from the nanoemulsion showed an initial burst release followed by a stable and controlled release for a longer period of 24 h. The nanoemulsion exhibited excellent thermodynamic and physical stability against phase separation and storage. The NAR-NE showed concentration-dependent cytotoxicity in A549 lung cancer cells, which was greater than that of free NAR. The percentage of apoptotic cells and cell cycle arrest at the G2/M and pre-G1 phases induced by NAR-NE were significantly higher than those produced by free NAR (*p* < 0.05). NAR-NEs were more effective than the NAR solution in reducing Bcl2 expression, while increasing pro-apoptotic Bax and caspase-3 activity. Therefore, stabilized NAR-NE could be a suitable drug delivery system to enhance the effects of NAR in the treatment of lung cancer.

## 1. Introduction

Lung cancer is the most common type of cancer worldwide with a high mortality attributed to diagnostic difficulties and the high potential to metastasize, the mortality associated with lung cancer is greater than that of prostate cancer, breast cancer and colon cancer, with a survival rate of approximately 15% [1,2,3]. The disease is highly heterogeneous and can develop in different locations of the lungs, thus resulting in highly unpredictable symptoms [1]. Lung cancer is classified into two major forms: non-small cell lung cancer (NSCLC) and small cell lung cancer (SCLC), with NSCLC being the most common [1]. Radiation and chemotherapy are the most usual treatments for lung cancer. Both strategies have various limitations, including the development of resistance, dose-related toxicity, lack of selectivity, and inadequate concentrations of the drugs in lung tissue as a result of increased interstitial pressure [4]. Thus, novel approaches with lower side effects and superior therapeutic efficacy are needed [5].

Both natural products and synthetic compounds are used to inhibit cancer development and/or to induce apoptosis in tumor cells [6]. These products include polyphenols, alkaloids, carotenoids, and nitrogen compounds, which are considered safe towards normal cells [6]. Naringenin (5,7-dihydroxy-2-(4-hydroxyphenyl)chroman-4-one or 5,7,4-trihydroxyflavanone) is a flavonoid mainly found in grapefruit and other citrus fruits; it has proven anti-inflammatory, anti-cancer, anti-mutagenic, anti-fibrogenic, and anti-atherogenic activities. In addition, this flavanone possesses free-radical scavenging properties [7]. Naringenin can induce cytotoxicity and apoptosis in cancer cell lines without causing toxicity in normal cells [7]. In A549 lung cancer cells, naringenin can increase TNF-induced apoptosis through enhancing death receptor-5 expression [7]. Moreover, it can act as a potent immunomodulator for the inhibition of lung fibrosis as well as metastasis [7]. Naringenin can also inhibit matrix metalloproteinases-2 and 9 in lung cancer [8]. It can stop cell proliferation and mobility by reducing the upregulation of phosphoinositide 3-kinase. Inhibition of cell proliferation and viability is due to inhibition of extracellular signal-regulated kinase 1/2 and upregulation of proliferating cell nuclear antigen (PCNA), nuclear factor B, and cytochrome P450 1A1 [7,9]. Naringenin treatment can activate p38 mitogen-activated protein kinase as well as the caspase 3 apoptotic pathway in cancer cells [10]. In liver hepatocellular carcinoma cells (HepG2), naringenin was found to produce mitochondrial dysfunction, an enhanced Bax/Bcl-2-expression ratio, as well as to induce apoptosis [11]. In cancer cells that expressed estrogen receptor-a/b, naringenin can cause apoptosis through activating p38 mitogen activated protein kinase and the caspase-3 pathway [10].

Naringenin has very limited water solubility (46 ± 6.0 μg/mL), gastrointestinal degradation, and low bioavailability, thus providing a major hurdle for its development as a drug and limiting its therapeutic use [12]. Several approaches have been undertaken to increase its solubility using nanotechnology [13,14,15,16,17]. Kumar et al. synthesized chitosan-encapsulated naringenin nanoparticles with antioxidant and anticancer activities [18]. Several naringenin-containing nanoparticulate systems have been developed; these include hyaluronic acid-decorated naringenin nanoparticles [7], silk fibroin nanoparticles loaded with naringenin [19], naringenin-containing poloxamer–chitosan-based nanoformulations [20], and chitosan nanoparticles containing naringenin [18]. Lipophilic drugs can be delivered orally in the form of nanoemulsions and this has been found to be one of the best approaches for the administration of naringenin, increasing its solubility and improving its permeation across the intestinal mucosa [21,22,23]. Nanoemulsion formulations can be effectively delivered through the oral route since they can enhance gastrointestinal absorption by increasing solubilization, extending gastric residence time, varying intestinal permeability, and stimulating the intestinal lymphatic transport pathway [24]. Nanoemulsions provide a potential drug carrier for treatment of cancer and are suitable for various administration routes, such as oral, parenteral, and topical [21]. Nanoemulsions are produced by the dispersion of two immiscible phases of nanometric sizes ranging between 20 and 200 nm [23]. The kinetic stability of nanoemulsions results from using a surfactant, or mixture of surfactant with a co-surfactant, allowing immiscible liquids to become miscible in a single phase by lowering the interfacial tension between them [23,24]. Naringenin nanoemulsions have been developed for the treatment of experimental Parkinson’s [23] and Alzheimer’s [25] diseases, but there have been few attempts to develop such nanoemulsions for the treatment of cancer. Sandhu et al. [26] developed a self-emulsifying nanosystem for the co-delivery of naringenin and tamoxifen, demonstrating its effectiveness on MCF-7 breast cancer cells, as well as its in vivo effectiveness in a rat breast cancer model [26]. In our present study we have developed a stable naringenin nanoemulsion that can significantly improve apoptotic activities as compared to free naringenin. In contrast to the work of Sandhu et al. we achieved this using a simple, natural naringenin nanoemulsion without the additional incorporation of any other anti-cancer agent. 

The objective of the present study was to develop and optimize a stable naringenin nanoemulsion for oral delivery, employing a Box–Behnken design. The effects of oil, surfactant, and cosurfactant concentrations on globule size were evaluated. The optimized nanoemulsion was tested for its thermodynamic stability and for its ability to release naringenin in vitro. Furthermore, the anticancer activities of the naringenin nanoemulsion and of free naringenin were investigated in A549 lung cancer cells; these activities were assessed by determining the effects on cell viability, mitochondrial membrane potential, and apoptosis, as well as by examining the effects on the cell cycle.

## 2. Results and Discussion

### 2.1. Solubility Studies

**Figure 1:**
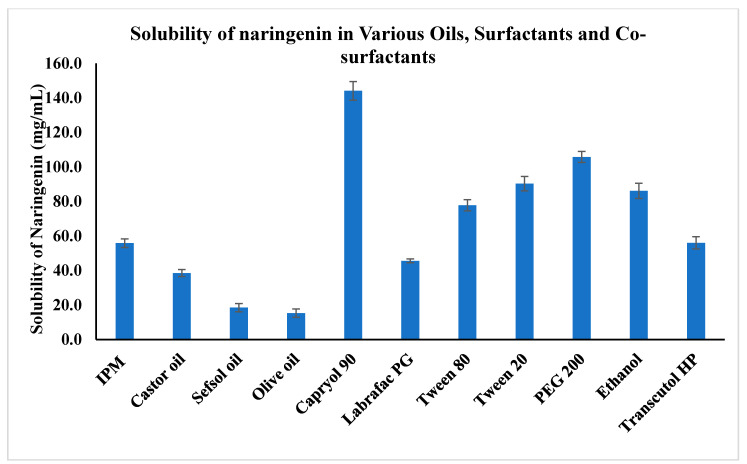
Solubility of naringenin in various oils, surfactants, and co-surfactants (*n* = 3) (*p* < 0.05; Capryol 90 vs. other oils, surfactants, and co-surfactants). Isopropyl myristate (IPM).

The solubility of pure NAR in an aqueous solution (49.82 ± 4.09 µg/mL) of 0.1 N HCl pH 1.2 (71.33 ± 3.40 µg/mL) and a phosphate buffer of pH 6.8 (90.32 ± 3.03 µg/mL) was determined. The poor solubility of NAR agrees with published data [12,16,27]. The solubility of naringenin in different oils, surfactants, and co-surfactants is shown in Figure 1. Naringenin was quite soluble in all the selected solvents but showed the highest solubility (144.09 ± 5.38 mg/mL) in Capryol 90 and the lowest in olive oil (15.24 ± 2.43 mg/mL), concurring with previous findings [25]. The second highest solubility (105.75 ± 3.19 mg/mL) was noted in polyethylene glycol 200 (PEG 200), in which naringenin was more soluble than in ethanol (86.11 ± 4.38 mg/mL); PEG 200 produced the highest solubility among the co-surfactants. Among the surfactants, Tween 20 was found to have the greatest effect on the solubility of naringenin (~90.30 ± 4.19 mg/mL). Based on these results, Capryol 90 was chosen as the oil phase. Further, the combination of Tween 20 and PEG 200 was chosen for the surfactant/co-surfactant mixture (Smix). Tween and PEG Smix systems are popular for micro- and nanoemulsions [28]. Similarly, the combination of Tween 20 and PEG 200 has also been reported as the Smix [29]. In the present study, the high solubility of naringenin in Capryol 90 was an advantage for the reduction of the oil concentration, allowing a lower concentration of Smix for the nanoemulsification process. The Tween 20 and PEG 200 combination could be considered powerful to emulsify and impart elasticity to the interfacial layer.

### 2.2. Formulation and Optimization of the Naringenin-Loaded Nanoemulsion

Based on the solubility studies, nanoemulsions were prepared with Capryol 90, Tween 20, and PEG 200. The data obtained for globule size for the chosen 15 formulations are given in Table 1.

#### 2.2.1. Mean Globule Size

The analysis of variance data show that mean globule size was significantly influenced by the concentrations of Capryol 90 (X1) and Tween 20 (X2) but not by the concentration of PEG 200 (X3) (Table 2). Further, the software indicated an equation for the calculation of globule size (Equation (1)).

Globule size = 397.69 − 22.9194X1 − 14.6557X2 + 7.97725X3 + 0.53055X1^2^ + 0.4932X1X2 − 0.334033X1X3 + 0.160117X2^2^ − 0.155333X2X3 + 0.02895X3^2^ (1)

The R-squared value was 96.6067%, while the adjusted R-squared (adjusted for degrees of freedom) was 90.4988%. The observed (actual) and fitted (predicted) values for the globule size are shown in Table 3. The observed and fitted values for the globule size were very close; the percent difference in their values, expressed as percent error, is very low for all the values.

**Figure 2:**
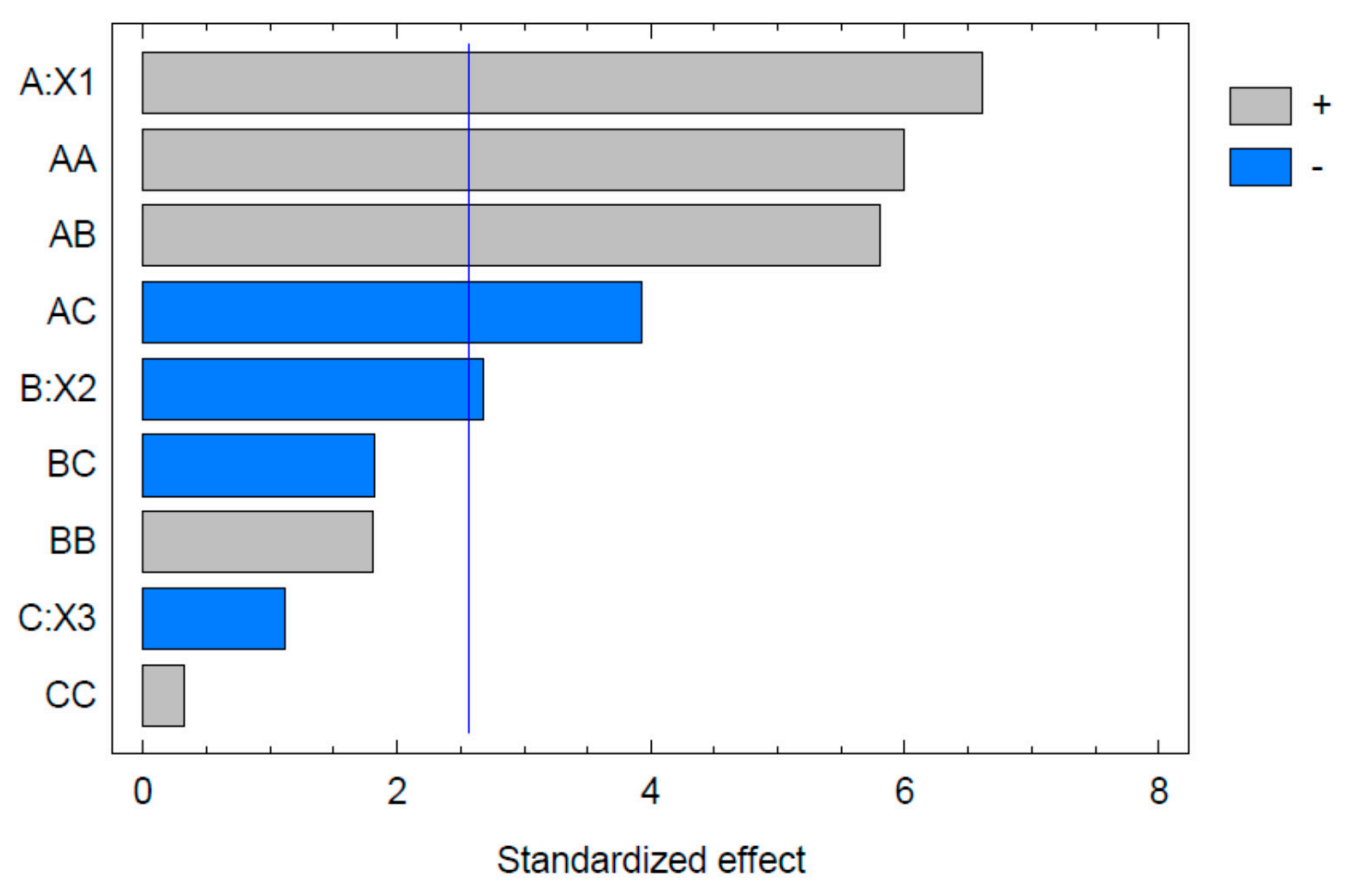
Pareto chart for globule size where X1 represent concentrations of Capryol 90, X2 represent Tween 20, and X3 represent PEG 200.

The Pareto chart obtained for globule size is shown in Figure 2. The significant factors were concentrations of Capryol 90 and Tween 20. This agrees with previous studies [30,31]. The oil, surfactant and surfactant-to-oil ratio are the factors with the greatest influence on the globule size of the nanoemulsions. It was further noted that the concentration of Capryol 90 had a positive effect, while Tween 20 had a negative effect on globule size; this means that increasing the concentration of Capryol 90 increases the globule size while the increased concentration of Tween 20 causes a lower globule size. These effects are explained in many previous reports [32]. Our results show that the co-surfactant concentration had little influence on the globule size. There are two explanations for this observation. Firstly, the role of the co-surfactant is to impart elasticity to the emulsifier film [32]; thus, rather than the concentration, the type or the ratio of surfactant-to-co-surfactant would be more important. Secondly, the range of concentrations studied for the co-surfactant was much less; in the present study, this range was only 5–15%. So, the effect of the co-surfactant concentration may not be completely understood when such a narrow concentration range is employed in the study. This could be rectified only if the effect of higher co-surfactant concentrations were to be examined. However, this type of study is not practically useful, as such concentrations are not applicable in nanoemulsions.

**Figure 3:**
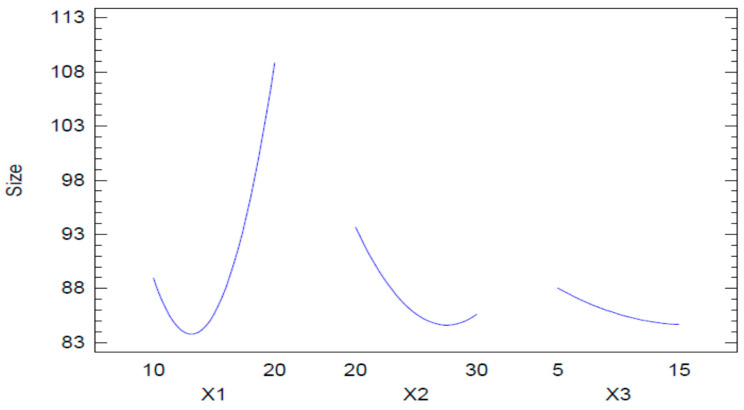
Main effects plot for globule size.

The results observed with the Pareto chart were also confirmed by the main effects plot for globule size (Figure 3). In this plot, it is seen that the concentration of Capryol 90 has the most influence on globule size. There was an initial, slight decrease in globule size, followed by a marked increase on further increasing the oil concentration. In the case of Tween 20, a marked decrease in the globule size was observed, although the effect was less than that of Capryol 90. However, at the higher concentration of Tween 20, the effect was less pronounced and even a slight increase in globule size was seen, although this effect cannot be considered important, as the change in globule size was negligible over the entire range of the concentrations used. A similar effect was observed with the co-surfactant. In this case, we can also see that the change in globule size was too small to reach a conclusion on its effect.

**Figure 4:**
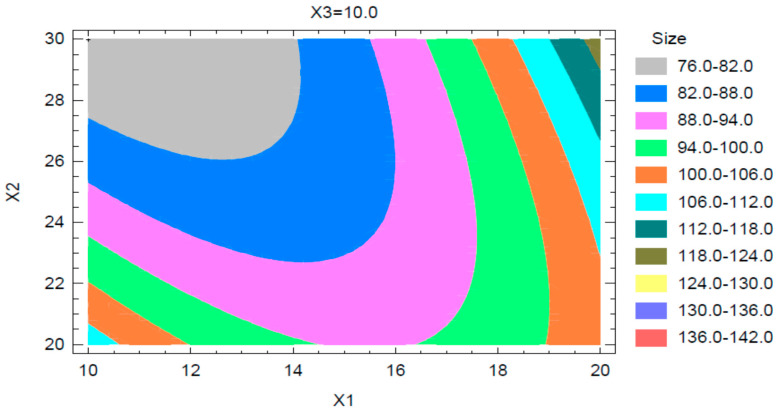
Contour plot for globule size.

The results and observations from the Pareto chart and main effects plot were further confirmed from the contour plot observed for the globule size. A sample contour plot is shown in Figure 4. This contour plot shows the interactions of concentrations of Capryol 90 and Tween 20, at a PEG 200 concentration of 10%, on the mean globule size of the nanoemulsion.

#### 2.2.2. Optimization of the Naringenin-Loaded Nano Emulsion (NAR-NE)

The optimized formula and the predicted responses are shown in Table 4. Minimum globule size was set as the goal in the software during numerical optimization.

### 2.3. Thermodynamic Stability

Nanoemulsions are thermodynamically stable systems, thus showing good physical stability and would be expected to pass all thermodynamic stability tests [23,25]. Indeed, the selected naringenin-loaded nanoemulsion successfully passed all the thermodynamic stability tests, with no phase separation, creaming, or cracking.

### 2.4. Characterization and Evaluation of the Optimized Nanoemulsion

#### 2.4.1. Mean Globule Size, PDI, and Zeta Potential

**Figure 5:**
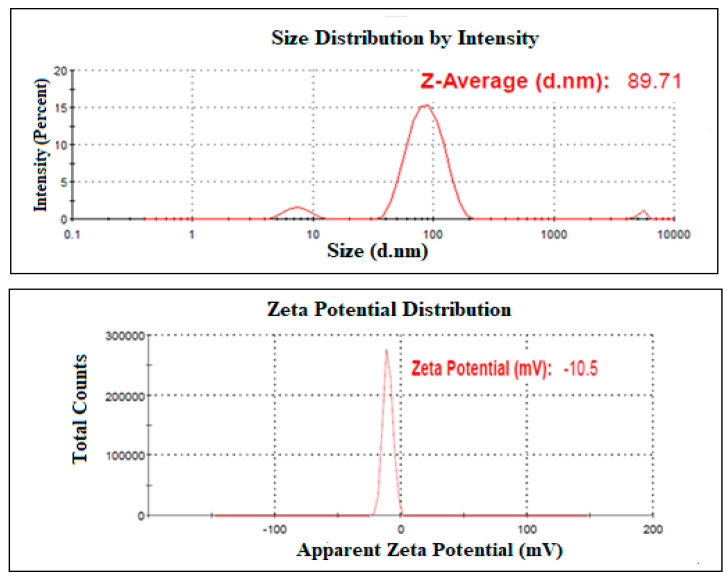
Representative data of globule size distribution and zeta potential of NAR-NE.

The mean globule size of the optimized NAR-NE was 85.6 ± 2.1 nm with a polydispersity index (PDI) of 0.263 ± 0.02 (Figure 5). The globule size was well in agreement with the predicted value. The zeta potential of the sample was −9.6 ± 1.2 mV (Figure 5).

#### 2.4.2. Transmission Electron Microscopy

**Figure 6:**
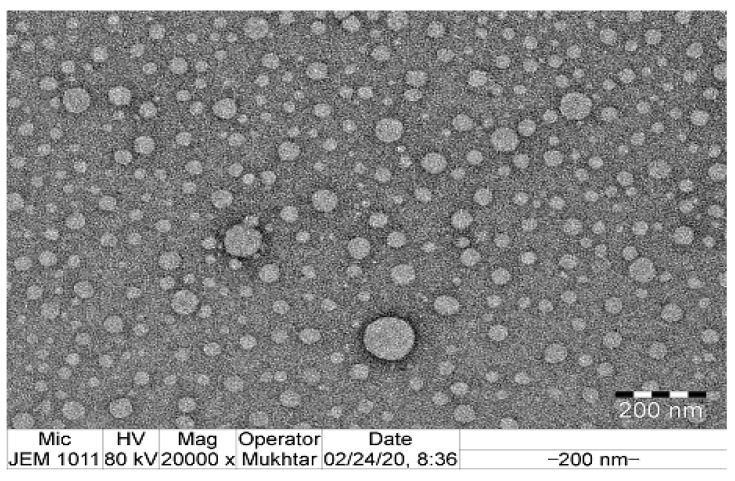
TEM image of the optimized naringenin nanoemulsion.

The morphology of NAR-NE globules was visualized from the TEM image (Figure 6). The observed result was well in agreement with the results observed for the mean globule size and PDI attained by photon correlation spectroscopy. From the image, the globules were found to be around 100 nm or less; they were homogenous and appeared spherical in morphology. The observed morphology was also comparable with reported nanostructured globules of naringenin and other drugs [23,25,33].

#### 2.4.3. Viscosity and Refractive Index

The optimized NAR-NE had a viscosity of 1.80 ± 0.72 mPa s, which could be used for oral and parenteral administration [34]. The refractive index was 1.279 ± 0.03, which confirmed the transparency of the nanoemulsion formulation. A very low value for the standard deviation of the refractive index was indicative of the homogeneity and the isotropic nature of the nanoemulsion [23].

#### 2.4.4. Percentage Transmittance and Drug Content

The percentage transmittance of the sample was found to be 96.49 ± 0.9%. This value was close to 100% and indicated a clear and transparent formulation. This further confirmed the homogeneity of the nanoemulsion formulation [20,21,23,25]. Such a response could be expected from the low globule size for the nanoemulsion. Any globule size less than one-quarter of the wavelength of visible light is expected to produce a transparent nanoemulsion [35]. The drug content was 97.34 ± 1.3% w/v of the nanoemulsion. A high drug content near to 100 indicates the stability of drugs during the preparation or nanoemulsification process.

#### 2.4.5. In Vitro Naringenin Release 

**Figure 7:**
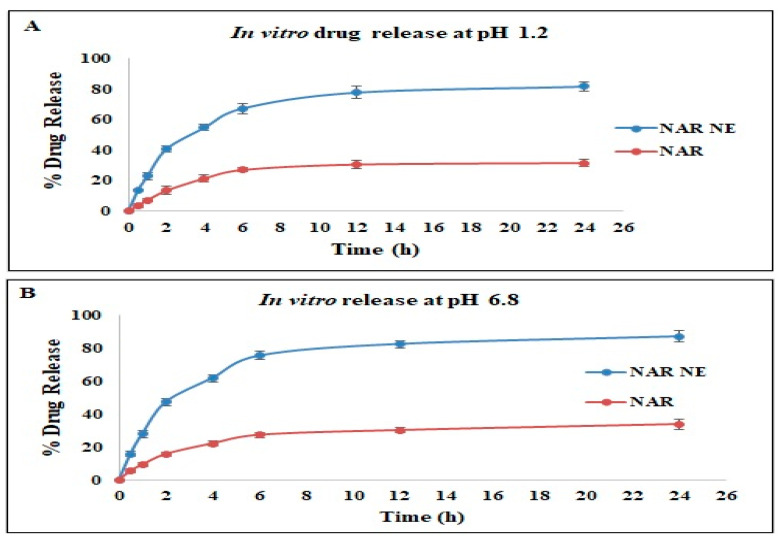
In vitro drug release study of naringenin (NAR) and a naringenin nanoemulsion (NAR-NE) in (**A**) pH 1.2 HCl and (**B**) pH 6.8 PBS. The data are shown as the mean ± SD, *n* = 3.

The results of the in vitro naringenin release study using the dialysis bag method is shown in Figure 7. Both the naringenin nanoemulsion and the naringenin suspension showed a biphasic drug release, as usually observed for these systems [22,36]. The NAR release from the nanoemulsion showed an initial burst release followed by a stable and controlled release for a longer period of 24 h. At all timepoints, there was a significantly higher release of naringenin from the nanoemulsion compared to the free NAR in the same release media. Nevertheless, there were no statistically significant differences between the release patterns for NAR-NE and free NAR in a pH 1.2 HCl solution and pH 6.8 PBS. The results indicated that the release behavior of NAR depends on the nanoemulsion formulation type and is independent of pH value [21]. A similar pattern for nanoemulsions has been reported elsewhere [21,22,36]. The drug release from the nanoemulsion is connected to the diffusion of droplets into the surrounding layers of surfactants in the buffer medium. The capability of nanoemulsions to impart rapid in vitro drug release is well established. Presentation of the drug in the molecular state, enhancement of the drug solubility, the presence and action of surfactants and co-surfactants, and the low droplet size are some of the accepted reasons for the better performance of the nanoemulsion formulation [21,22,37].

#### 2.4.6. Stability Studies

**Figure 8:**
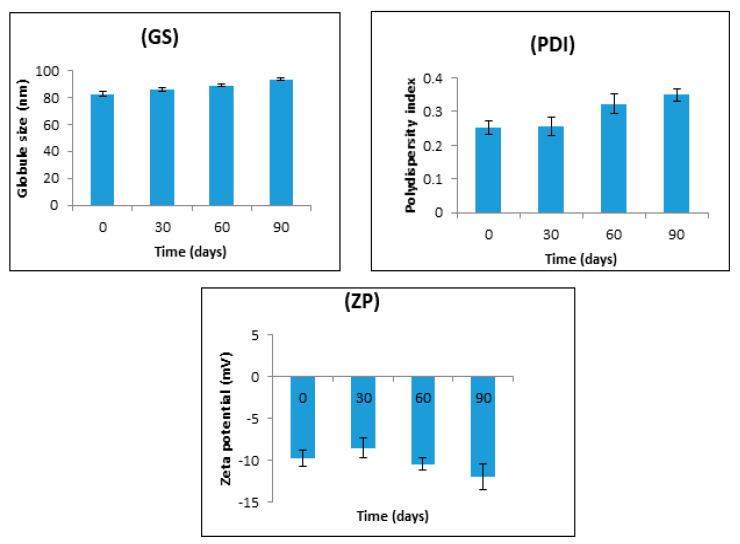
Effect of storage for up to three months at 40 ± 2 °C and 75 ± 5% RH on the globule size (GS), polydispersity index (PDI), and zeta potential (ZP). The data are presented as the mean ± SD, *n* = 3. The slight increase in particle size, PDI, and ZP was not significant (*p* > 0.05) for adjacent sampling points.

The results of stability studies carried out at 40 ± 2 °C and 75 ± 5% RH for three months are shown in Figure 8. All the studied parameters remained within an acceptable range throughout the three-month study period. The mean globule size shows a slight increase (82.51 ± 1.88 to 93.67 ± 1.27 nm) on storage; this very small change is normal even in the most stable nanoemulsions and is insufficient to influence their stability [22,38]. Similar observations were also noted in the case of PDI and zeta potential. The PDI increased slightly from 0.253 ± 0.22 to 0.350 ± 0.019, this being possibly related to the slight increase in the mean globule size of the nanoemulsion. There was also a slight increase in the zeta potential (−9.78 ± 0.97 to −11.94 ± 1.52 mV) on storage at 40 ± 2 °C and 75 ± 5% RH for 3 months. Previous studies have reported a decrease in the magnitude of zeta potential [39].

### 2.5. Cell Viability Using MTT Assay

**Figure 9:**
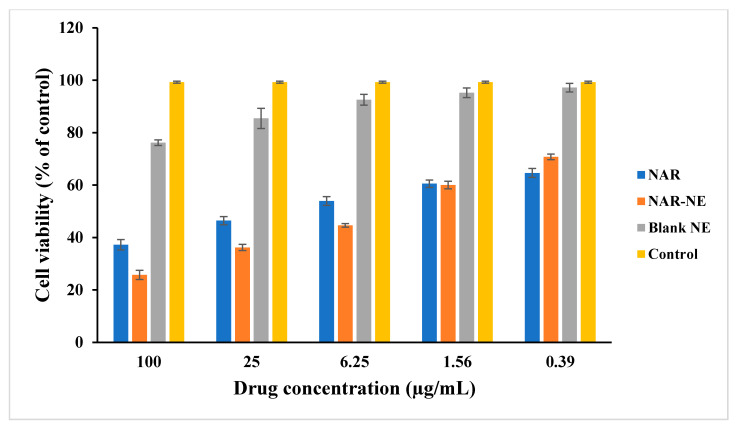
MTT results of in the vitro cell viability of the control group, free NAR, blank NE, and NAR-NE against A549 cells incubated for 24 h. The data are presented as the mean ± SD (*n* = 3). *p* < 0.05 for free NAR and NAR-NE at 6.25, 25, and 100 µg/mL.

The MTT assay showed a concentration-dependent reduction in cell viability for the NAR-NE and free naringenin (NAR) with respective IC_50_ values of 19.28 ± 3.21 and 37.63 ± 7.27 µg/mL (Figure 9). NAR-NE produced the lowest cell viability (25.75 ± 1.75, 36.19 ± 1.18, and 44.62 ± 0.68%) at 100, 25, and 6.25 µg/mL, respectively, in comparison with NAR (37.21 ± 1.98, 46.44 ± 1.55, and 53.93 ± 1.69%). The greater potency of the naringenin nanoemulsion relative to free naringenin may reflect the higher release of naringenin from the nanoemulsion [26,40]. The difference in effectiveness between the nanoemulsion and free naringenin was much more marked at higher concentrations (100, 25, and 6.25 µg/mL). Similar enhancements of the cytotoxicity of NAR-NE formulations in a concentration-dependent manner was observed by other researchers [41]. The blank nanoemulsion did not show substantial cell cytotoxicity at 25 µg/mL. However, there was a decrease in cell viability at 100 µg/mL (76.19 ± 1.09), which could be due to the presence of surfactants and co-surfactants in the formulation.

### 2.6. Effect of the Naringenin Nanoemulsion on Inducing Apoptosis as Detected Using the Mitochondrial Membrane Potential Method

**Figure 10:**
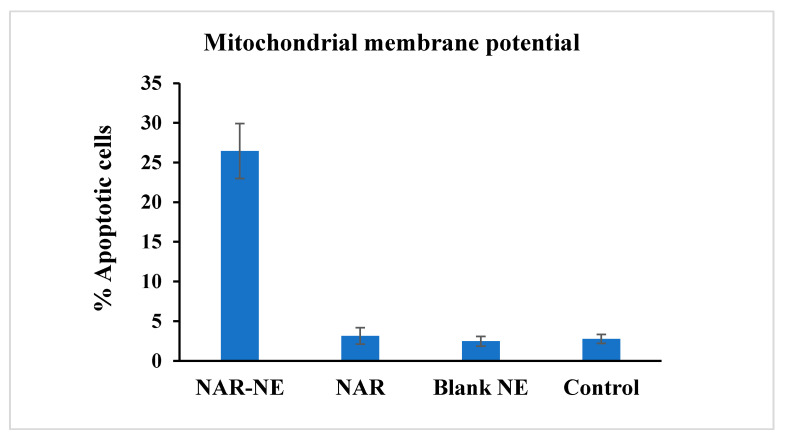
Effect of the free NAR, blank NE, and NAR-NE (IC_50_ concentrations) in producing apoptosis in A549 cancer cells, as determined by measurement of mitochondrial membrane potential (*p* < 0.05 for free NAR vs. NAR-NE).

Treatment with NAR-NE showed the highest percentage apoptotic cells (26.44 ± 3.46%) compared to NAR (3.15 ± 1.03%), the blank (2.48 ± 0.62%), and the control (2.76 ± 0.57%) (Figure 10). The higher apoptosis and anti-cancer activity of the naringenin nanoemulsion was probably due to the longer contact time and better availability of naringenin from the nanoemulsion compared to the free drug [26,40,42].

### 2.7. Apoptosis as Determined Using the Annexin-PI Method

**Figure 11:**
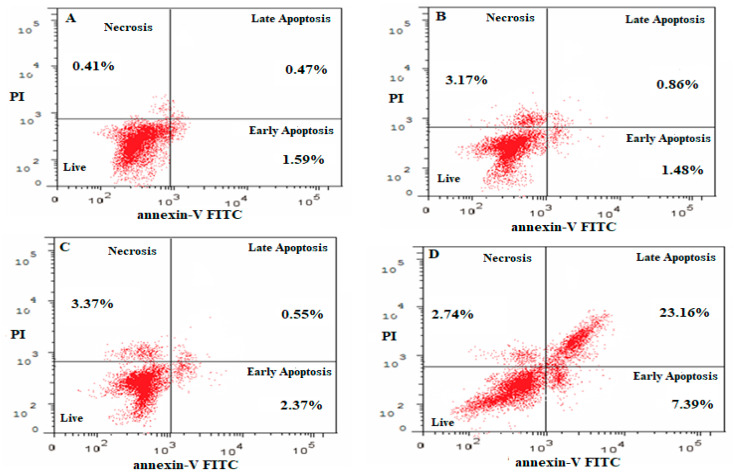
Flow cytometry results using the Annexin V and PI assay showing the distribution of cells in untreated and treated A549 cells: (**A**) control; (**B**) blank NE; (**C**) NAR; (**D**) NAR-NE.

**Figure 12:**
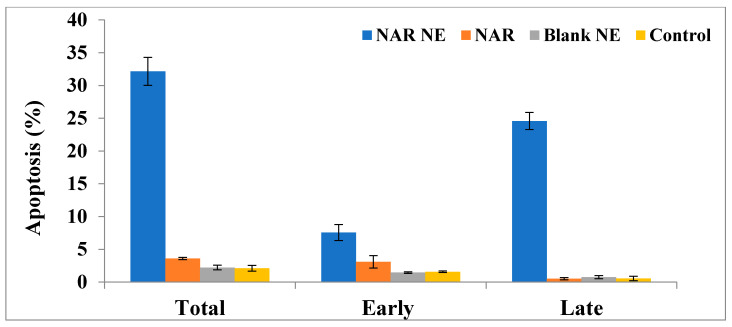
Effect of the IC_50_ concentrations on free NAR, blank NE, and NAR-NE (compared with no treatment) in inducing total, early, and late apoptosis in A549 cells (*p* < 0.05 for free NAR vs. NAR-NE).

The effect of free NAR and NAR-NE on inducing apoptosis was determined using the Annexin-PI method and flow cytometry, as shown in Figure 11 and Figure 12. In the absence of naringenin, the percentage of cells showing apoptosis was very low for the control (2.12 ± 0.44%) and blank NE (2.22 ± 0.35%) (Figure 12). Treatment with NAR-NE produced a significant increase in total apoptotic activity compared with the free NAR treatment (32.16 ± 2.14% versus 0.52 ± 0.17%, respectively; *p* < 0.05). This difference may result from the low solubility of the naringenin in the culture media, as solubility determines the contact time between the anti-cancer agent and the cancer cells [42,43]. These findings support the potential anti-cancer activity of naringenin and the ability of the nanoemulsion system to enhance its dissolution in vitro.

### 2.8. Cell Cycle Analysis by Annexin-PI Method

**Figure 13:**
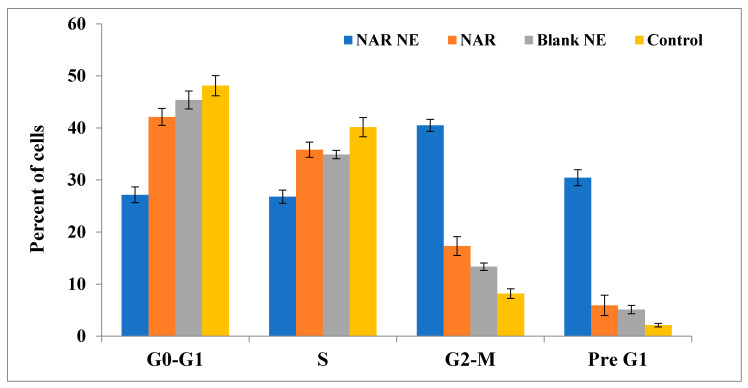
Cell cycle analysis of A549 cells treated with IC_50_ concentration of free NAR, blank NE, and NAR-NE (*p* < 0.05; free NAR vs. NAR-NE).

The cell cycle analysis shows the effects of the different formulations on the cell cycle phases and provides an understanding of the molecular mechanisms of cell cycle arrest (Figure 13). NAR is known to cause apoptosis in lung cancer cells by decreasing the AKT activity and inhibiting matrix metalloproteinases-2 and 9 in A549 cells [8]. Treatment with NAR-NE produced a substantial increase in the proportion of apoptotic cells in the pre-G1 (30.45 ± 1.54%) and G2/M (40.48 ± 1.15%) compared with the blank and free NAR (*p* < 0.05) (Figure 13). Treatment with free NAR slightly increased the proportion of cells in the pre-G1 (5.93 ± 1.96%) phases and G2/M (17.32 ± 1.79%) compared with the blank formulation (*p* > 0.05). Accumulation of many cells in the pre-G1 and G2/M phases is a distinguishing feature of apoptosis. This indicates that the effect of the nanoemulsion on activating apoptosis is related to an increased proportion of cells at the pre-G1 phases and cell arrest at the G2/M phase. Similar findings were observed by others [7,41,42,43].

### 2.9. Effect of the Naringenin Nanoemulsion on the Expression of Bax and Bcl-2 Proteins

**Figure 14:**
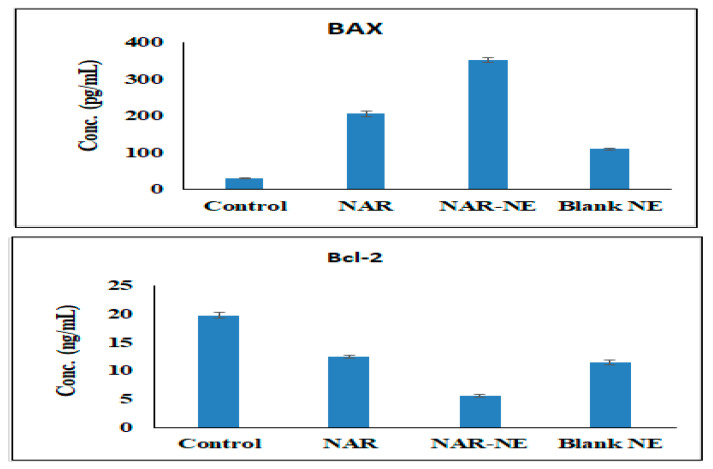
Effect of the IC_50_ concentrations of free NAR, blank NE, and NAR-NE on the expression of Bcl-2 and Bax protein in A549 cancer cells (*p* < 0.05 for free NAR vs. NAR-NE).

Expression of Bax protein induces apoptosis, while expression of Bcl-2 enhances oncogenic/anti-apoptotic activities (Figure 14) [40,42]. Treatment with NAR-NE produced a significant (*p* < 0.05) increase in the expression of Bax (351.40 ± 6.31 pg/mL) compared with free NAR (206.20 ± 8.04 pg/mL); this may be explained by the enhancement of dissolution produced by the nanoemulsion. On the other hand, treatment with NAR-NE produced a marked decrease in the expression of Bcl-2 compared with the control, with the effect of the nanoemulsion being greater than that of free naringenin [40,42].

### 2.10. Effect of the Naringenin Nanoemulsion on Caspase-3 Activity in A549 Cells

**Figure 15:**
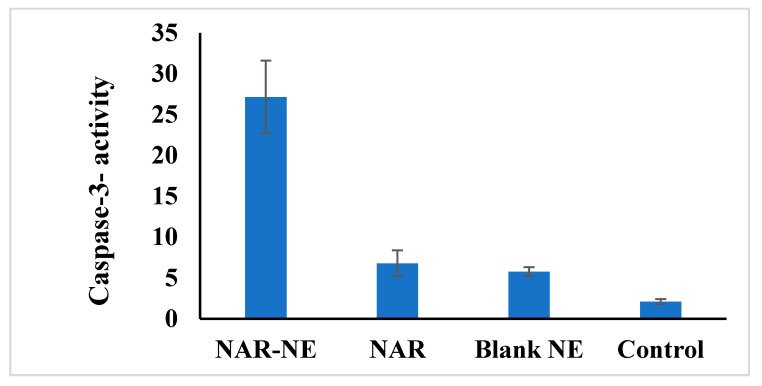
Effect of treatment with IC_50_ concentrations of free NAR, blank NE, or NAR-NE on caspase-3 activity in A549 cancer cells (*p* < 0.05 for free NAR vs. NAR-NE).

Caspase-3 activity is associated with cancer cell apoptosis [23]. Treatment with NAR-NE produced a significant (*p* < 0.05), 4–5-fold increase in caspase activity compared with free NAR [41] (Figure 15). Again, the increased effectiveness of the nanoemulsion relative to the free drug may relate to the enhanced availability of naringenin. A critical requirement of the drug delivery systems is their ability to release the drug in an appropriate concentration at the target site in its active form.

## 3. Materials and Methods

### 3.1. Materials

Naringenin, isopropyl myristate, castor oil, and olive oil were purchased from Sigma-Aldrich, (St. Louis, MO, USA). Ethanol, Tween 20, Tween 80, and PEG 200 were purchased from Merck, USA. Sefsol 218 was procured from Nikko Chemicals (Tokyo, Japan), whereas Capryol 90, Transcutol HP, and Labrafac PG were obtained from Gattefosse (Saint Priest Cedex, France). The Caspase-3 Colorimetric Assay Kit (Catalog No. K106) was purchased from BioVision, USA. Human Bax ELISA (EIA-4487) was purchased from DRG International, Inc., USA. The Bcl-2 ELISA Kit (Cat. No. 99-0042) was procured from Invitrogen Corporation, CA, USA. The Thiazolyl Blue Tetrazolium Bromide (MTT) reagent kit was purchased from ABCAM, Cambridge, UK. The Annexin V-FITC Apoptosis Detection Kit and Cell cycle kits were purchased from BD Pharmingen (San Diego, CA, USA). Other chemicals used were of analytical grade.

### 3.2. Cell Culture

Adenocarcinomic human alveolar basal epithelial cell (A549) lung cancer cells were purchased from ATCC (Manassas, VA, USA). A549 cells were cultured in a Roswell Park Memorial Institute (RPMI) 1640 Medium supplemented with 10% fetal bovine serum (FBS), penicillin, and streptomycin. The cell line was grown at 37 °C under a humidified atmosphere with 5% CO_2_ to 80–90% confluence.

### 3.3. Solubility Studies

The studies to determine the solubility of naringenin were performed in distilled water, 0.1 N HCL (pH 1.2), a phosphate buffer (pH 6.8), as well as in various oils, surfactants, and co-surfactants, as described previously [25,44]. Naringenin was added in excess to 2 mL of the sample under study in Eppendorf tubes. The sample was subjected to vortexing for 15 min and then kept in a shaking water bath for 72 h [44]. The sample was finally centrifuged, and the supernatant was dissolved in a definite volume of methanol. The naringenin content was determined by UV spectrophotometry at 289 nm after suitable dilution with methanol [25].

### 3.4. Formulation and Optimization

#### 3.4.1. Experimental Design

The optimization of the naringenin-loaded nanoemulsion was undertaken using a Box–Behnken design [45], as shown in Table 1. Concentrations of Capryol 90 (X1), Tween 20 (X2), and PEG 200 (X3) were chosen as the independent variables and mean globule size (GS) was taken as the response. The design was generated and evaluated using Statgraphics software (Statgraphics Technologies, Inc., Warrenton, VA, USA).

#### 3.4.2. Formulation of the Naringenin-Loaded Nanoemulsions

The naringenin powder was dissolved in Capryol 90. To this sample, a mixture of Tween 20 and PEG 200 was introduced and vortexed for 30 min for proper mixing. The sample was then permitted to reach equilibrium for 48 h at room temperature. The specified amount of water was then added in small portions with sufficient vortexing after each addition [25].

#### 3.4.3. Optimization of the Naringenin-Loaded Nanoemulsion

The nanoemulsion was optimized by the numerical method [45]. Minimum values for the responses (globule size) was set as the goal in the software during numerical optimization. The optimum formula suggested by the software was further prepared, characterized, and evaluated.

### 3.5. Thermodynamic Stability 

The thermodynamic stability of the optimized nanoemulsion was assessed by the method of Kotta et al. [46]. Centrifugation, heating–cooling cycle (HCC), and freeze–thaw cycle (FTC) tests were carried out (Table 5). 

### 3.6. Characterization and Evaluation of the Optimized Nanoemulsion

The optimized naringenin nanoemulsion was tested for globule size, polydispersity index (PDI), and zeta potential after 100 times dilution. The parameters were assessed using a Zetasizer (Nano ZSP, Malvern, Worcestershire, UK).

#### 3.6.1. Transmission Electron Microscopy (TEM)

A TEM image was recorded after appropriate dilution of the nanoemulsion. Negative staining (phosphotungstic acid, 2%) was carried out after placing one drop of the sample, after suitable dilution, over a copper grid, whereupon the images were captured (JEOL, JEM 1010, Tokyo, Japan; 60–80 kV).

#### 3.6.2. Viscosity and Refractive Index

The naringenin-loaded nanoemulsion was tested for its viscosity with a viscometer (Model DV-E, Brookfield, Middleboro, MA, USA). The refractive index was recorded using a refractometer (Abbe’s type). The recordings were carried out in triplicate.

#### 3.6.3. Percent Transmittance and Drug Content

For the determination of percent transmittance, the sample was analyzed in a UV–Vis spectrophotometer at 650 nm [23]. For the determination of naringenin content in the nanoemulsion, 1 mL of the NAR–NE was prepared. A total of 100 µL of NAR-NE was taken out and diluted with 10 mL of methanol. The diluted methanol solution was passed through a 0.45 μm membrane filter and the percentage NAR content in the NE was finally analyzed using a UV–visible spectrophotometer at 289 nm [25,44].

#### 3.6.4. In Vitro Naringenin Release

The in vitro drug release study was undertaken using a dialysis bag technique [21,44]. The molecular weight cut-off of the dialysis bag used was 12kDa. The release medium consisted of 900 mL of a pH 1.2 HCl solution or pH 6.8 phosphate buffer set at 37 ± 2 °C. The dialysis bags were filled with a 2 mL sample of the NAR suspension and a NAR-NE equivalent to 10 mg of pure NAR. The filled dialysis bags were tied and a weight of 1 g each was attached to each bag with thermoresistant thread to hold it within the release medium, and introduced into a dissolution test apparatus (USP, Apparatus 2, Paddle type) set at 50 rpm. Samples of 1 mL were withdrawn at different timepoints, namely, 0.5, 1, 2, 4, 6, 12, and 24 h, and replaced with the same volume of dissolution medium to maintain the sink condition. The drug release was determined at 289 nm by UV–VIS spectrometry in triplicate [47].

#### 3.6.5. Stability Studies

The optimized naringenin-loaded nanoemulsion batch was prepared in triplicate and stored at 40 ± 2 °C and 75 ± 5% RH for three months. Samples were withdrawn at 1, 2, and 3 months after starting the test. Initial samples (Time = 0 months) were also analyzed. The samples were analyzed for visual appearance. The samples were also subjected to mean globule size, polydispersity index, and zeta potential determinations [22].

### 3.7. Cell Viability Determination Using MTT Assay

The A549 cells were seeded in 96-well plates at a density of 5 × 10^3^ cells/well and were treated with 0.39, 1.56, 6.25, 25, and 100 μg/mL naringenin as the free active material, NAR-NE, as well as the blank NE for 24 h. Then the cells were treated with 10 µL of a 5.0 mg/mL MTT solution and again kept for 4 h incubation at 37 °C. After washing with phosphate buffer saline (PBS), the precipitates were dissolved in 150 µL DMSO for 20 min and absorbance readings were taken at 563 nm. Comparison of absorbance of each precipitate at 563 nm was used to determine the relative cell viability with vehicle treated/control groups for each concentration [42,43].

### 3.8. Mitochondrial Membrane Potential 

Mitochondrial membrane potential was measured by using a tetramethylrhodamine methyl ester (TMRM) assay kit in which TMRM was used as the probe. The A549 cells were allowed to grow in a 96-well plate with a cell density of 1 × 10^5^ cells/well and treated with different formulations (NAR-NE, NAR, blank-NE, and the control group). The treated cells were washed with PBS after 24 h of exposure, stained with a TMRM working solution at 37 °C, and incubated in the dark for 30 min. Finally, the TMRM solution was removed and the cells were washed again with PBS and analyzed using a flow cytometer [43].

### 3.9. Apoptosis Determined by the Annexin V-Propidium Iodide Method 

To determine the apoptosis induced by NAR, NAR-NE, the blank, and control media, the Annexin-V staining technique was used. The 1 × 10^5^ cells/well were seeded in 6-well plates and incubated with IC_50_ concentrations of the formulations for 24 h (37 °C) and then collected and centrifuged at 200× *g* for 5 min. The harvested cells were then washed twice and re-suspended in PBS at room temperature. The Annexin V-FITC (10 µL) and propidium iodide (PI) solution (5 µL) were added to the mixture and incubated for 5 min (25 °C). The analysis was carried out using a flow-cytometer (FACS Calibur, BD Bioscience, CA, USA) [40,42,43].

### 3.10. Cell Cycle Analysis by the Annexin-PI Method

For the determination of cell cycle distribution, A549 (1 × 10^5^ cells/well) were seeded and incubated with the NAR, NAR-NE, blank, and control group for 24 h. After 24 h of incubation, the medium was extracted and washed with PBS twice, trypsin was added, and the cells were centrifuged. The pellet was rinsed with PBS, fixed in 70% ethanol, and then kept at −20 °C overnight. Before analysis, the cells were rinsed with PBS to remove the alcohol and stained with PI and RNAase as per the manufacturer’s instructions and analyzed using flow cytometry [40,42,43]. 

### 3.11. Bax and Bcl-2 Proteins Expression Estimation

Bcl-2 and Bax protein expressions were analyzed in all samples by enzyme-linked immunosorbent assay (ELISA). This was undertaken using monoclonal antibodies against Bax (Ab-1) (DRG^®^ Human Bax ELISA Kit) and anti-Bcl-2 (Zymed^®^ Bcl-2 ELISA Kit) according to the manufacturer’s instructions.

### 3.12. Caspase-3 Analysis

The caspase-3 analysis was performed using the Caspase-3 Colorimetric Assay Kit (BioVision, Milpitas, CA, USA). A549 cells were seeded (3 × 10^6^ cells/well) and treated with the NAR, NAR-NE, blank, and control, were re-suspended in chilled lysate buffer, and the then cell lysate was incubated on ice for 10 min prior to centrifugation (10,000× *g* for 1 min). The test procedure for the caspase-3 assay was performed as per the manufacturer’s instruction and the color was measured at 405 nm using a microplate reader [43]. 

### 3.13. Statistical Analysis

Data were represented as the mean ± standard deviation (SD) of three independent experiments. The statistical significance was calculated using Student’s t-tests and one-way analysis of variance (ANOVA) with a Tukey post-hoc test. Data with *p* values < 0.05 were considered significant.

## 4. Conclusions

The Box–Behnken design was used to optimize the naringenin-loaded nanoemulsion formulation. Mean globule size was found to be significantly affected by concentrations of Capryol 90 and Tween 20, whereas the zeta potential was dependent on the concentration of Tween 20 alone. The optimized formulation passed the thermodynamic stability testing. The globule size, PDI, zeta potential, viscosity, refractive index, percentage transmittance, and drug content of the nanoemulsion were within the acceptable values. The in vitro drug release from the nanoemulsion was significantly higher than free naringenin. The accelerated stability studies confirmed the physical stability of the nanoemulsion. The globule size, PDI, and zeta potential remained to be acceptable without any significant change after storage at 40 ± 2 °C and 75 ± 5% RH for three months. These results indicated that a nanoemulsion formulation of naringenin provides a stable system with high drug release. NAR-NE showed higher cytotoxic, apoptotic cell death, and cell arrest both at the G2/M and sub-G1 phases when compared with free NAR in A549 cells. The NAR-NE was more effective than free NAR in increasing caspase-3 and the pro-apoptotic Bax protein, while reducing the expression of the anti-apoptotic protein Bcl2. These findings suggest that the stable NAR-NE can provide a suitable drug delivery approach for the treatment of lung cancer. However, in vivo animal studies are required to confirm the therapeutic potential of NAR-NE in lung cancer therapy.

## Figures and Tables

**Table 1:** Independent variables with the coded values, the dependent variable, and the responses obtained for the various formulation trials.

Run	Values of the Independent Variables			Dependent Variable
	Factor X1 (% *v*/*v*)	Factor X2 (% *v*/*v*)	Factor X3 (% *v*/*v*)	Mean Globule Size, GS (nm)
1	15	25	10	85.63
2	20	25	15	103.07
3	20	20	10	105.16
4	10	30	10	75.98
5	15	20	15	92.39
6	10	25	5	79.47
7	10	20	10	110.69
8	20	25	5	116.78
9	15	25	10	85.63
10	20	30	10	119.77
11	15	25	10	85.63
12	10	25	15	99.17
13	15	30	5	96.09
14	15	20	5	94.38
15	15	30	15	78.57
**Independent Variable Factor**		Levels		
		Low (−1)	Medium (0)	High (1)
Concentration of Capryol 90 (X1)		10%	15%	20%
Concentration of Tween 20 (X2)		20%	25%	30%
Concentration of PEG 200 (X3)		5%	10%	15%

**Table 2:** Analysis of variance data for globule size.

Source	Sum of Squares	Df	Mean Square	F-Ratio	*p*-Value
A:X1	789.435	1	789.435	43.68	0.0012
B:X2	129.659	1	129.659	7.17	0.0439
C:X3	22.7925	1	22.7925	1.26	0.3125
AA	649.577	1	649.577	35.94	0.0019
AB	608.116	1	608.116	33.65	0.0021
AC	278.946	1	278.946	15.43	0.0111
BB	59.1631	1	59.1631	3.27	0.1302
BC	60.3211	1	60.3211	3.34	0.1273
CC	1.93408	1	1.93408	0.11	0.7568
Total error	90.371	5	18.0742	---	---
Total (corr.)	2663.23	14	---	---	---

**Table 3:** Data for the actual and fitted values of mean globule size.

Run	Mean Globule Size (nm)	
	Observed Values	Fitted Values
1	85.6333	85.6333
2	103.073	99.5158
3	105.16	104.53
4	75.98	76.6104
5	92.3933	96.5812
6	79.4667	83.0242
7	110.693	109.322
8	116.777	119.593
9	85.6333	85.6333
10	119.767	121.138
11	85.6333	85.6333
12	99.1667	96.35
13	96.0933	91.9054
14	94.3767	92.1904
15	78.5767	80.7629

**Table 4:** Optimized nanoemulsion formula and predicted responses.

Factor		Optimized Formula/Predicted Response
Independent	X1 (%)	15.7801
	X2 (%)	30.0
	X3 (%)	15.0
Dependent	GS (nm)	83.2564

**Table 5:** Details of the thermodynamic stability testing.

Test	Conditions	Evaluation Parameters/Criteria
Centrifugation	5000 rpm; 30 min	Cracking; Creaming; Phase separation
*HCC*	4 °C and 40 °C; 48 h in each temperature; 3 cycles	
FTC	−20 °C and 25 °C; 48 h in each temperature; 3 cycles	
